# A day in the life of people with severe mental illness living in supported housing

**DOI:** 10.1186/s12888-020-02896-3

**Published:** 2020-10-15

**Authors:** Carina Tjörnstrand, Mona Eklund, Ulrika Bejerholm, Elisabeth Argentzell, David Brunt

**Affiliations:** 1 Department of Health Sciences, Occupational Therapy and Occupational Science, Lund University, Box 157, SE 221 00 Lund, Sweden; 2 Department of Health and Caring Sciences, Linnaeus University, Växjö, Sweden

**Keywords:** Activity, Psychiatric disabilities, Social environment, Occupational therapy, Supported accommodation

## Abstract

**Background:**

People with severe mental illness (SMI) living in supported housing (SH) struggle in everyday life and we currently lack a comprehensive body of knowledge concerning how the residents experience their day. This paper aimed to gain knowledge about how people with SMI describe a day in SH in Sweden, in particular the activities they most frequently engage in and how they experience what they do in or outside their home. Furthermore, it is important to gain knowledge of which activities motivate residents to leave the housing facility and to participate in the community. This new knowledge can help staff to encourage a recovery process among the residents.

**Methods:**

One hundred thirty-three people living in SH completed a time-use diary and a mixed-methods approach was applied, including calculations of what activity that was most frequently performed and a manifest content analysis addressing experiences of activity.

**Results:**

The residents had a low activity level and were often alone. Approximately one-half of the reported activities were performed in their own apartments, and generally unaccompanied. A quarter of the activities were performed in the common areas and a further quarter outside the SH. The most frequently performed activities were quiet and tranquil ones, e.g. listening to music and resting. Doing errands and group activities with staff and residents were the main activities that motivated leaving the facility. The participant experience of a day is presented in three categories: “Experiences of chosen and enforced togetherness and overcoming loneliness”, “Environmental change and emotional balance can generate activity”, and “Met and unmet needs for support, friendship and security”.

**Conclusions:**

The residents were generally satisfied with their quiet and tranquil lifestyle and appeared to demand little of life, which may relate to previous experiences of institutional life and can constitute a challenge for staff. The findings highlight experiences that can help to improve SH. Services need to support individually adjusted contextual stimuli and individualize the support to help residents find a good balance and motivate them to be active in and outside SH, which can support a recovery process.

## Background

Supported housing facilities emerged in Sweden and abroad after de-institutionalization as a way to support individuals with severe mental illness (SMI) who had difficulties in coping with the demands of everyday life in the community [1]. One of the basic aims of the mental health reform in Sweden, which took place in the mid-1990s, was to improve the living conditions and quality of life for this group. This was done by transferring the location of care from an institutional environment to residential facilities in the community. The environment in supported housing in Sweden has, however, been criticized for its institutional nature. Similarities between supported housing and institutional care, such as a lack of personal freedom and enforced togetherness, were found by Brolin, Brunt, Rask, Syren and Sandgren [2], who highlighted the importance of moving towards a supportive environment that can enhance self-determination. Only a few studies have been performed in the Swedish context and none of these have focused on activities in the everyday life for individuals in supported housing. Recent reviews [1, 3] have also showed that research on these aspects in supported housing is scarce.

Two types of municipal housing facilities have become the most common alternatives in Sweden: small congregate facilities, termed supported housing (SH), and ordinary housing with outreach support services [4]. Approximately one-third of those with SMI in Sweden live in SH and generally have a private room or apartment, whilst sharing common areas with other residents and staff. These housing facilities have been found to generate a sense of security [5, 6] as well as providing the residents with easy opportunities for socializing [5, 7]. On the other hand, the close proximity of other residents and staff has been experienced as an enforced togetherness that can limit integrity and privacy [8, 9], while Brolin et al. [2] found that the main concern of those living in SH was being deprived of self-determination.

Meaningful activities in a person’s everyday life are important for the recovery process [10, 11] and a balance between activities is important for the person’s health and well-being [12]. The surrounding community offers many opportunities for meaningful activities that could bring about positive change. Nordaunet and Sælør [11] showed that being active in a social context where one experiences a positive identity was seen to generate meaningful activities and be a central influencing factor in the recovery process.

A general conclusion of research into the everyday activities of people with SMI is that most of them are under-stimulated [13] and that the disability itself can prevent a balanced and satisfactory time use [14]. Moreover, they have often lacked the opportunity to establish a pattern of daily habits and routines and have thus found difficulty in organizing their daily activities [15]. It is common for people with SMI to have a low level of engagement, although this can vary between individuals [16]. Furthermore, they may not take initiatives to plan or participate in activities despite having housing support. This thus can entail that they do not gain the type of experiences that could stimulate individual development and motivation.

A number of factors can influence how people with SMI perceive their activities, these include personal factors such as the severity and manifestations of the illness, which can have a significant impact on motivation and the ability to carry out everyday activities. Another significant factor is the environment where people with SMI spend a majority of their time. The home environment can constitute a double-edged sword that in one sense provides security and a place to rest, while on the other hand has been found to be restrictive for this group [17].

Day centers, with either a social or work orientation, are another environment of importance for SH residents. These are frequently visited by people with SMI in general and provide opportunities for meaningful daily activities, companionship and a daily structure [15, 18, 19]. However it was reported in a Swedish study that only a few of the participants who attended day centers with a work-orientation lived in SH [18].

Research into the activities of people with SMI living in the community has generally focused on the activities they perform without focusing on their housing situation as a specific variable [20]. Most studies appear to have been carried out among those who live independently in ordinary housing. A recent study, however, showed that individuals living in supported housing reported that they had too little to do and were under-occupied [21], indicating a need to further explore this situation. There appears to be a knowledge gap concerning the activities of those living in SH; what they do in or outside their home, what they engage in and how they experience what they do during a day. Such knowledge may contribute to the literature about SH residents and the development of adequate support for them to engage in meaningful activities.

The aims of this study were thus to describe which type of activities people with SMI living in SH perform, the frequency of these activities, where these activities take place and how they are experienced. A further aim was to investigate which activities motivate SH residents to leave the housing facility and to participate in activities in the community.

## Methods

A convergent mixed-method design with both qualitative and quantitative methodology was applied in order to gain a comprehensive view of the activities performed in and around supported housing facilities [22].

### Study context

SH in Sweden is a congregate residential facility where residents have been assessed by a social worker as needing more support than is generally provided in ordinary housing with outreach support. Staff support can vary from office hours to 24 h per day.

The accommodation in SH can either consist of a fully-fitted apartment in a housing block with similar apartments, including one for in-house staff with facilities for group activities, or a bedroom and sitting room together with communal bathroom and eating facilities. SH in Sweden generally have 5–12 places but larger facilities do exist. The SH included in the study were situated in both urban and suburban areas. Some of the residents had opportunities to go out into a garden, while others only had a balcony. All of the SH facilities were situated close to parks or nature areas.

Residents in SH facilities in Sweden receive counselling and treatment at the community mental health centers. Moreover, the staff in SH provide psychological support and are available for discussing everyday problems as well as being supportive in activities such as eating, shopping, doctors’ appointments and physical activities. SH facilities are mainly staffed with a variety of qualifications to meet the residents’ needs with a predominance of nursing assistants, but also social workers, behavioral scientists and recreational instructors. Nurses came to the housing units on a daily basis, but often had more than one SH to attend to, and an occupational therapist could come when called for.

All of the SH facilities in this study had a staffed common area in the same or an adjacent building. The availability of a private kitchen varied between facilities, and communal cooking areas were used for food preparation in some of the facilities. The provision of food also varied, where some SH offered the choice of having one or all meals served, while others had a policy of not providing cooked food but instead offered support with cooking or the reheating of ready-made food.

### Selection procedures

Nineteen strategically selected municipalities in Sweden were invited to participate in this study, two of which were major cities with self-governing districts. The aim was to obtain variations in terms of geographical location (southern, western, middle and eastern Sweden), smaller towns and larger cities and sociodemographic characteristics (proportion of Swedish/foreign born citizens, more/less affluent areas, central/suburban areas). Twenty-one of the 27 municipalities/districts, which were invited to take part, accepted and a total of 35 SH units participated. Each unit contained 5–10 apartments. After approval from the managers of the housing support services, each SH unit was contacted, and an information meeting was held in each unit. Staff asked the presumptive participants and research assistants were then introduced and provided both oral and written information. A gift card worth 100kr ($10) was given to each of the participants in recognition of their participation after signing an informed-consent form.

### Data collection procedure

Eleven research assistants (including the first author), nine of whom were occupational therapists with experience of working with people with mental illness and two with a university degree and experience of data collection with the study’s target group, were enrolled for the data collection. All the research assistants received training on how to administer the instruments and how to perform the data collection. The data, which was part of a larger study, was collected 2014–2016 and a more in-depth exploration of the quantitative data is presented in a related study by Eklund et al. [21].

The residents, who agreed to participate, were contacted by the research assistants to book a time and a place of their choice for the interview. This was generally held in their own apartment but could also be in the common or staff office areas. The participants were informed of the voluntary nature of participating and were offered breaks to counteract tiredness, and assistance if necessary. Between two and seven residents agreed to participate from each of the 35 units. Twenty-two (of 155) individuals were unable to complete the diary due to not being able to recollect what had occurred the day before, not wanting to share the information, having difficulties understanding the relevance, or being too tired.

### Instruments

#### Background questionnaire

A questionnaire focusing on background data included questions about sociodemographic factors, self-reported diagnosis, activity level and motivation for activity. Questions such as “how often do you go out?” and “what motivates you to go out?” were asked in order to gain further information about the participant’s activity level and motivation. The self-reported diagnosis was classified by a psychiatrist, according to the ICD-10 system [23].

#### Profiles of occupational engagement

The residents in SH were invited to describe the day (24 h), prior to the data collection. The time-use diary interview that is part of the POES (Profiles of Occupational Engagement in people with Severe Mental Illness) [16, 24, 25] instrument was used for this description. The participants were also asked whether it was considered a normal day. A data collection sheet representing 1 day was completed by each participant. The sheet was divided into five columns: when the activity was performed (hour by hour), the type of activity, with whom (if any) the activity was performed, where the activity took place, and finally personal reflections on the activity. The research assistants assisted the participants in completing the time-use diary during the interview by using prompting questions to fill gaps, e.g. such as “Do you remember what you did before lunch?”, while at the same time being careful not to influence its content. Table 1 shows an example of the time use diary.

**Table 1:** Example from a section of a diary and narrative format

When activity was performed	What type of activity	With whom it was performed	Where the activity took place	Personal reflection on the activity
One hourly intervals	*What did you do?*	*Was there anyone else around at the time?*	*Where were you at the time?*	*How did you experience the activity?*
	Record everything that you did and try to remember and note down for how long the activities were performed.		Name the place and location.	
		Record whether you were with someone or not.		Record your personal reflections and comments
***Two selected rows from a diary:***				
1 pm – 2 pm	Accompanied a friend to buy a stereo	A friend	In town	Could not find anything
2 pm - 3 pm	Ate	alone	At McDonalds	Relaxed, better than sitting alone in my room.
	Drank cider		Outside X (a pub)	

An example of the amount of information a diary could contain transferred to narrative format (the same participant as above)

At midnight, I watched TV. Did not go to bed until two o’clock in the morning. I had trouble falling asleep. I woke up at 11 am feeling rested. Checked the email on the computer. Ate nothing, I find it hard to eat in the morning. At 1 pm, I went into town with a friend, he was going to buy a stereo, but could not find anything. I ate at McDonald’s at two o’clock. Bought cider and sat outside X (a pub) and relaxed, it’s better than sitting alone in my room. At 6 pm I was at a friend (at the SH), listening to music, “old earrings”, nice in some ways, better than being home alone. At nine o’clock I watched TV at home alone, did not have the energy for anything else, mostly crap on TV. (10725)

### Data analysis

#### Quantitative analysis

The activities reported in the diaries were divided into three groups to correspond to the study aim: Firstly what the participants did (the type of the activity), secondly where they performed this activity (the geographical location) and thirdly with whom (the social environment). Each of these groups was sub-divided to provide detailed information; for example, the sub-classifications in the geographical location were: own apartment, other resident’s room, common area in SH and outside SH. Activities were grouped together to form a type of activity with a similar nature. For example, cleaning, tidying up, washing clothes etc. were termed “household chores”. The number of times these activities occurred, the geographical location and the social environment were then summed – see Table 4. The frequencies of activities that motivated the individual to go out, which were reported in the background questionnaire, were calculated. This quantitative part of the study entailed a simple calculation of the number of times an activity was mentioned in the diaries, and not the actual time used. A calculation of time use would be an interesting method to apply in a future study but was not possible with the data collection in the present study. The socio-demographic data (Table 3) were calculated in terms of means and percentages using SPSS 25.0.

#### Qualitative analysis

A manifest qualitative content analysis of the diary content was performed [26]. Content analysis is considered to be a suitable method when analyzing the manifest content of data [27] as found in the five columns of the POES diaries. The columns in all the diaries were overviewed prior to the start of the analysis and it was seen that some of the participants had found it difficult to remember details about their day and needed prompting questions. For example, the ability to recall what time activities took place, and in what order, varied greatly among the participants and it was difficult for the researchers to attain a clear grasp of the content as the research assistants’ notes were not always in exact chronological order in the diaries. To enhance the rigor of the study, and after reading all the diaries, a consensus decision was thus made to process the diaries into a short narrative format (shown in the final section in Table 1) as has been done in previous research [16]. This thus provided the researchers with a more comprehensive picture of what a day in SH could look like for the participants, rather than the disjointed pieces of information in the row and column structure of the diary. The narratives were transcribed by the first author and two research assistants after consensus as to how this transcription process was to be performed.

The narratives were read independently by the main author (an occupational therapy researcher) and the last author (a psychiatric nursing researcher) prior to a joint discussion regarding the main impression of the content areas in the diaries: activities, the social environment, the geographical location and reflections about the activities. Meaning units were extracted from the narrative text and were, in accordance with Graneheim and Lundman [26], condensed step by step. Examples of the analysis steps are presented in Table 2. The abstracted meaning units were then labelled with a code (e.g. what was said about the environment when reflecting on the “common areas” and “meal times” and what the participants experienced during activities) and then sorted into sub-categories and categories. The categories created in this process refer to an explicit content of text. The participants’ positive and negative reflections on experiences in each category were incorporated in the sub-categories and categories [27, 28]. The first and last authors, utilizing their different professional perspectives, reached consensus during all the steps of the analysis process (abstracted meaning units, condensation and reasoning about positive and negative reflections). Consensus was also reached about the three categories and seven sub-categories, which represent the participants’ experiences of their day in the SH environment. The other co-authors discussed and agreed to this analysis on several occasions during the process. Short quotations from the narrative form of diaries are used to exemplify the categories and sub-categories in the findings.

**Table 2:** Example of analysis steps

Meaning Unit	Condensed meaning unit. Close to the text	Condensed meaning unit interpreting the underlying meaning	Sub-Category	Category
“then listen to music in the day room, reading and exchanging magazines with others, it feels good …, we eat dinner together in the dining room and it’s nice. After dinner, I meet another resident in her apartment.”//	Importance of social contact and activity together in the SH. Eating, sharing and helping each other was experienced positive.	Living together with others invites sharing, helping and social contact and motivates activity	Sharing common areas meant having friendship nearby and overcoming loneliness.	The SH context gave experiences of both chosen and enforced loneliness and togetherness
“Some residents steal, others are ok.”	There were difficulties living close together.	Enforced togetherness can bring difficulties	Living close to others was experienced as enforced togetherness	

## Results

### Participants

A total of 155 people with SMI living in SH agreed to participate in the study and 133 (86%) completed the diary. A majority of those reporting their diagnosis had some form of psychosis. The sociodemographic characteristics of participants are presented in Table 3.

**Table 3:** Participant characteristics

	Living in SH *N* = 133
Mean age; mean (SD)	48 (12)
Proportion of men	56%
Single	99%
Years in current housing; mean (Range)	6 (0.1–32)
Born in Sweden	86%
Has children	24%
Has a friend	80%
Attends day centre	50%
-Education	
-Not completed 9-year school	5%
-Completed 9-year school	48%
-Completed high school	36%
-Completed college/university	10%
Self-reported diagnostic category	
-Psychosis	50%
-Anxiety/mood disorders	11%
-Other (Asperger syndrome, ADD or unspecified mental disorder	18%
-Missing data	21%

#### Quantitative data

The analysis of the frequencies of the performed activities revealed that a majority of residents generally reported one single activity for the morning, the afternoon and the evening in their diaries. However, there were many blank spaces indicating no reported activities. Just over 50% of the number of activities were performed in their own apartments and then mostly alone. Approximately a quarter of all activities were performed in the common areas of the SH together with other residents and staff. A further quarter were performed outside of the supported housing facility, either alone or with others (Table 4). The two most common activities performed within the home environment of their own apartment involved calm and tranquil activities, such as listening to music/radio, watching TV and resting, or the more active pastime of TV-games. Shopping and doing errands were the main activities outside the SH, which is confirmed by these being named as the main motivator (31.8%) for leaving the SH. Exercise in the form of walking, cycling and swimming was the second main motivator (28.1%) for going out. Further motivators were; group activities/DC/work rehabilitation (12%), Socializing (9.9%), coffee/eating out (5.2%), treatment (3.6%) and unspecified reasons (9.4%). One hundred and one participants stated that they went outside every day, 21 went out once or twice a week, two said they never went out and nine did not respond to the question about how often they went out.

**Table 4:** Number of most frequently reported activities performed, where and with whom

Type of activity		Geographical location – where		Social environment – with whom	
**Inside Supported Housing**					
Listening to music, radio, TV, film, TV-games, reading	**117**	Own apartment	318	Alone	**276**
Resting, sleeping (daytime), brooding	**80**			Other resident	**8**
Preparing meals	**63**			Staff -	**16**
Household chores	**29**			Others (friend – unspecified if it was a resident, relative, priest)	**18**
Socializing	**19**				
Hobby	**10**				
Companionship (TV, coffee, music, card games)	**135**	Common area in SH	**135**	Other residents and staff	**135**
Socializing	**7**	Other resident’s room	**7**	Other resident	**7**
**Outside supported housing**					
Shopping, Errands	**58**	Outside SH	**162**	Alone	**65**
Group activities (DC, courses, trips)	**37**			Staff and other participants (DC, course, trips, work training)	**42**
Walk/Cycling	**28**			Friend/relative	**29**
Eating out	**20**			Staff	**24**
Treatment	**12**			Other resident	**2**
Gym	**5**				
Church	**2**				
**Total amount of activities**					
Activities – total sum	**622**	Inside SH Sum	**460 (74%)**	Alone	**341 (54.8%)**
		Outside SH Sum	**162 (26%)**	Group activities together with staff/residents or other participants	**177 (28.4%)**
				Friend/Relative	**47 (7.6%)**
				Staff	**40 (6.4%)**
				Other resident	**17 (2.7%)**

#### Qualitative data

The manifest content of the qualitative data showing the residents’ experiences of everyday activities in the SH context formed three categories and seven sub-categories. The categories were: *Experiences of chosen and enforced togetherness and overcoming loneliness*, *Environmental change and emotional balance can generate activity*, and *Met and unmet needs for support, friendship and security*.

### Category: experiences of chosen and enforced togetherness and overcoming loneliness

The residents’ experiences represented two clearly contrasting perceptions; the proximity to other residents could generate togetherness and friendships in the sharing of common spaces and of common experiences of difficulties in everyday life, which could also help them to overcome loneliness. On the other hand, the proximity to other residents could also entail having to live close to someone you feared or just did not like being with.

Three sub-categories could be identified: ‘Sharing common areas led to having friendship nearby and overcoming loneliness’, ‘Living close to others was experienced as enforced togetherness’ and ‘The importance of being given a choice to participate or to be alone’.

#### Sub-category: sharing common areas led to having friendship nearby and overcoming loneliness

The residents spoke of the importance of friendships with other residents and that being with them was an important part of their day. Routines were created around doing activities together with other residents and staff or inviting residents into one’s own apartment.

The most frequently used way to overcome loneliness was by being in the common areas with staff and other residents and to a lesser extent in each other’s apartments. This generated positive feelings of togetherness through being able to talk to others or listen to music and read or exchange magazines with other residents. The common spaces in the SH provided opportunities for socializing both indoors and in the garden area.

Eating together was the most frequently reported social activity (approximately one third of the residents were served food), which was appreciated as a “nice and normal” routine. Socializing in terms of celebrating birthdays together and having the opportunity to share something with another resident, such as “watching TV with a friend”, “talking and listening to music together” or “hanging out with residents in the garden” were positive experiences.

#### Sub-category: living close to others was experienced as enforced togetherness

The close proximity to other residents also generated negative experiences due to problems with uninvited guests, rowdy people in the common areas or problems with drugs and theft. The enforced togetherness at meal times was perceived as causing “difficulties eating with others” being as “everyone has their problems”. Other unpleasant situations in the common areas were when other residents intruded on one’s private space by demanding cigarettes, food, money or companionship. Solutions were found, however, by “eating alone and then joining the others” or “pairing up with someone because we both have difficulties socializing”. Another example is through the development of a friendship as described in the following quote; “one of the residents, a drug addict, disturbs the others and is aggressive. I feel scared to death. However, I stay in the common room and a person I know massages my legs which feels nice…. we watch television and chat”.

#### Sub-category: the importance of being given a choice to participate or to be alone

There were examples of being able to participate in cooking teams or baking sessions, if the residents wanted to cook and eat with others. Being able to choose group activities or individual, staff-assisted activities was seen as positive and being allowed to decline to participate was important.

The physical environment of the SH units varied, and not all of the participants had a fully equipped kitchen in their own apartment and did thus not have the opportunity to prepare meals or be more self-dependent in preparing food. The meal service at SHs in one municipality had been withdrawn as the result of a policy change to encourage the residents to go shopping and prepare their own meals. This activity demanded help and training from staff and often residents either had to wait for staff to have time to help them or find other solutions. In spite of the potentially positive effects of this policy change in terms of developing cooking skills and greater self-dependency, some residents only bought ready-made food, which was experienced as “boring” and “it feels lonely eating on your own”. Several residents valued the opportunity to be able to choose what to eat, where to eat it and whether they were to eat alone or not, while a lack of a choice was often experienced negatively.

Being able to choose loneliness and inactivity was thus also important and having one’s own apartment helped in this regard; “having your own place to withdraw to and be alone” and to feel that “it’s nice to have nothing to do”. Being able to be alone, which many of the residents valued, could on the other hand also be seen as difficult when they had not actively chosen to be alone.

### Category: environmental change and emotional balance can generate activity

The participants’ diaries demonstrated how they alternated between periods of activity to find stimulation and periods of withdrawal in order to feel better. Being among others could be experienced as too stressful for longer periods, and the environment within their own homes inspired quieter and more peaceful activities such as listening to music or watching TV. Leaving their own homes and venturing out into the community or the SH’s common areas inspired them to be more active.

Two sub-categories could be identified: ‘Needs, routines and helping others spurred activities outside one’s home’ and ‘Using activities to regulate feelings’.

#### Sub-category: needs, routines and helping others spurred activities outside one’s home

The activities that motivated the residents to leave their homes were commonly initiated by needs and routines. The need to purchase something could encourage the residents to go over the threshold and cycle or walk to the nearby grocery shop; “I had not been out in a few days. I had no coffee at home, must have coffee in the morning. I also bought cigarettes and ice cream”.

This initial activity could also have a spin-off effect of meeting a friend, just as walking past the bike stand could stimulate the “feeling to cycle a bit and take a ride to McDonalds”. Other important stimuli to be active outdoors were routines such as “eating in a neighbor’s apartment”, “feeding carrots to the rabbits in the garden”, “working at a day center” and “bringing out the trash”. Moreover, helping each other and being needed by friends, an animal, other residents or staff at SH were major motivators for being active, for example “assisting with shopping”, “getting help washing up”, “cleaning the apartment together”, “getting help withdrawing cash”, “going to agencies together” or “serving at dinner” (in the common area).

#### Sub-category: using activities to regulate feelings

The residents alternated between periods of calmness and tranquility or low-intensive physical activities, while periods of more stimulating activity appeared to have the effect of regulating feelings. It was as though the informants tried different techniques to cope with and understand feelings of stress, symptoms or anxieties. There seemed to be a need for calming activities that entailed withdrawing from contextual stimuli, which most commonly entailed resting alone and listening to music or the radio or watching TV.

Listening to music or the radio was spoken of as a positive experience and a way to cope when one needed “to feel in control” or a way to distract hallucinatory voices and to help “against compulsive behavior”. Moreover, it was a relaxing activity that had a calming effect and could also enable meditation while listening. These activities were also perceived as “a way to pass time” or to lose track of time by “watching TV 24/7”.

However, finding the right emotional balance when using activities to regulate feelings appeared to be difficult and there were expressions of ambivalence. For example, resting could “feel good but also sad” and walking could “feel positive but also fearful and stressful” and coming home could “feel both pleasant and difficult”.

### Category: met and unmet needs for support, friendship and security

Staff provided support for all types of problems and for initiating activities and were regarded as friends, someone to be with and a stable person when the resident felt ill and needed someone to talk to. The diaries demonstrated that the staff had difficulties in balancing active support and passive presence and offering the right amount of support in activities.

Two sub-categories could be identified: ‘Staff helped to break loneliness and were someone to confide in’ and ‘The residents’ needs for support were not always met in the way the residents wanted it’*.*

#### Sub-category: staff helped to break the residents’ feelings of loneliness and were someone to confide in

The diaries provided several examples of the interaction with staff and the support and stimulation given by them. The resident had someone to talk to and confide in when he/she was troubled but also felt that the staff could be like a friend in helping to motivate and to initiate activities. The residents spoke of going to the staff to “get in a good mood again” after feeling “under-stimulated, both intellectually and socially”. It felt positive when the staff initiated activities, such as just walking outside and picking flowers, after long periods of rest in bed. The staff were also regarded as conversational partners and “it felt nice to be with the staff on your own, to talk about private things” and being with the staff was a way to “feel safe and to break the negative thoughts”.

The residents also met staff at other community services, for example, some residents went regularly to day centers (49%) and spoke to staff there as a way of breaking their loneliness. The diaries also showed that leisure activities such as excursions, group activities and “Friday-night-movies” were positive and were further examples of staff-led activities seen as a way of breaking the residents’ loneliness.

#### Sub-category: the residents’ needs for support were not always met in the way the residents wanted it

Based on the residents’ reports, it appeared that the staff had difficulties in managing to create a correct balance in terms of giving the right amount of support at the right time, and when the balance was wrong the support could be felt as degrading or confusing.

Moreover, limitations in the environment such as staff shortages, or residents not having their own kitchen, affected the resident’s opportunity to get the support they needed, such as “we had to wait for each other and cook when the staff were ready to help”. The residents reported a wide variety of problems in everyday life that they needed help with and felt that the staff sometimes focused more on practical issues than psychological and emotional ones and one resident spoke of being forced to clean when “I would rather have talked a while first”. If the staff helped too much when the residents were ill, staff assistance could be seen as belittling; “it’s nice to do as much as possible yourself, and a pain when I felt downgraded by staff that helped me”.

## Discussion

### Being active in a limited social and physical context

The results showed that the participants reported low levels of activity in a limited social and physical environment. They experienced activities with friends or staff as motivating and positive even though their social environment was limited, and the common area played a major part in the individuals’ activity repertoire and social context. This latter finding appears to concur with a recent literature review showing that services may benefit from a more multifaceted approach, giving attention to both psychosocial interventions and environmental circumstances [3, 29].

The type and nature of the activities performed by the residents, as presented in Table 4, have elements that are similar to those that are commonly found among the general public, such as: the preparation of meals, household chores, relaxation through music and television, and outdoor activities such as shopping, eating out and walking. The major differences appear, however, to be that the residents with SMI generally lack leisure activities and/or a workplace to go to, have an emphasis on companionship with others in a similar situation, perform solitary activities in the home and are generally less active. The latter concurs with the findings of Bejerholm and Eklund [17], who showed that people with SMI had a low activity level, with often quiet and tranquil activities such as resting, watching TV, listening to the radio or just pacing around, that were performed alone in the home. However, withdrawing and carrying out such activities, or simply not doing anything, also appeared to have a regulatory effect on the participants’ emotions by generating a feeling of calm, which could also reduce stress and the intensity of external stimuli. These forms of ‘non-doing’ may be seen as constituting a natural part of a recovery process from an occupational viewpoint [30] or a way of coping with overwhelming environmental stimuli [31]. It thus becomes important for staff to ascertain how the residents themselves experience this apparent inactivity, since it is common for professionals to view the lack of activity among people with SMI in mainly negative terms [32].

Two external environmental aspects are of significance for occupational engagement in this context. These are the geographical location, i.e. where the activities took place and the social environment, i.e. the people with whom the activities were performed. Most activities reported in the diaries took place indoors, in the participant’s own apartment, and were carried out alone. This result is alarming as it has been shown in research that it is important for people with SMI to be in a social context, which can provide a positive identity [11, 19], can increase the individual’s ability for self-determination [2, 33] and can provide opportunities for individually chosen meaningful activities [11, 18].

The common indoor and outdoor areas of the SH were important for providing opportunities for meals and joint activities and overcoming loneliness by offering companionship with staff and other residents there as shown in the study by Dorozenko et al. [6]. This also corresponds to the research by Roos et al. [5], who demonstrated the importance of having both a fully private equipped apartment and shared communal areas.

Another aspect of the geographical environment concerns how the physical environment is equipped, which could influence both the level and the nature of the residents’ activities. For example, a fully equipped kitchen in the apartments in SH could increase the possibilities for greater self-dependence among the residents. The significance of the physical environment in SH units has been focused on in research by environmental psychologists [34], who found that SHs with greater possibilities for social interaction, privacy and restoration had larger numbers of and a greater quality of interactional behavior among the residents. A number of studies have shown that the physical structure of a building has a major impact on the residents’ relationships with other residents. For example, SH facilities have generated a stronger sense of belonging and companionship for the residents than they have previously experienced when living alone [35–37].

### Individually adjusted contextual stimuli to motivate residents to be active

Apart from needs related to shopping, the study showed that the main contributor to venturing outside was group activities with staff or other residents. In order for the residents to engage more in activities outside the home, the staff may need to act in a coaching role prior to, during and after the activity. This can be done by explaining in detail what is to be done, to adapt the environment during the activity, and to confirm that the individual has done something well afterwards. This type of approach is well described in the remotivation theory [38], and the use of this theory in interventions has shown positive results in earlier studies with both depressed and cognitively low functioning individuals, not greatly dissimilar to the target group in the present study [39–41]. An implication for SH services can be to stimulate the development of a recovery nurturing environment as described by Nordaunet & Sælør [11]. Furthermore, service managers may need to consider more education for residents and SH staff [42], as well as day center staff in order for them to act as motivators for supporting the residents’ personal recovery and for reducing their loneliness.

### Freedom of choice, goals and power-sharing

An important aspect of the residents’ activities in the present study was the opportunity to decline to participate, which is a theme found in many studies in the SH context. Brolin et al. [2] reported how residents spoke of having limited options for influencing their own living situation, which was linked to the nature of the housing form and led to feelings of powerlessness. In a Danish study, self-determination entailed having influence on daily activities, which was perceived as valuable together with having rehabilitation goals [43]. Goalsetting in combination with autonomous motivation and empathic others has shown to be successful. Moreover, Krotofil, McPherson & Killaspy [3] pointed out that the residents’ ability to make choices and exercise agency was contingent on the relationships between residents and staff and the ethos and principles behind the service provision. SH services that enable power-sharing between staff and residents and co-produced support and goal setting are warranted [2, 5, 33]. Furthermore, shared decision-making has been shown to be an important aspect of recovery-oriented services [44–46].

### Regulation of feelings and environmental stimuli

The results also showed that the residents had difficulties finding the right balance in many aspects of life in the SH facilities and their stories also indicated that staff had similar difficulties in balancing their support. The residents appeared to have a repertoire of contrasting actions, which they used to regulate feelings; these actions included being alone or being together with others, being passive and resting or being active. Similarly, the residents’ stories indicated that the staff seemed to have to balance the provision of active support with being passively present.

Finding the right pattern and number of activities, shown to be important for wellbeing and health [12] appeared to be a constant struggle for the residents. For example, doing something with someone else could be desirable but also entail a worry about not being able to regulate feelings, and the residents appeared to have difficulties in balancing their emotions and expressed an ambiguity resembling anxiety that could lead to them avoiding activities and social interactions. The need to balance feelings through being passive or active has been maintained as leading to avoiding situations that can entail a great deal of stimuli, unless one is able to easily withdraw to solitude [31]. This has thus implications for both staff and residents. The staff in SH services need to gain knowledge about environmental stimuli and their effect on the residents, while the residents may need to learn more about regulating their own reactions to stimuli, as described by Champagne [47] which might enhance their activity repertoire and reduce their loneliness. Furthermore, identifying significant others who can help bridge the fear of overstimulation [31] and be a safe supporter in a new environment can be important [38, 39].

The preparation and consumption of food constitutes one of the main activities in SH facilities and is a further example of environmental stimuli. This activity includes elements such as the daily need for food, the procedures for its preparation, the standard and equipment level of the kitchen environments, and the inherent social aspects of living close to others. These elements constitute a major influence on the level of the residents’ activity, albeit in different ways. For those preparing food themselves, the purchase of ingredients and the cooking provided opportunities for activity and maintained or increased independence and satisfied a basic need, whilst also being a solitary activity. On the other hand, for those who ate their meals together with other residents and staff in the common areas of the SH, the activity sufficed the basic need for food, provided companionship while not generating opportunities for independence and improving cooking skills. The routines at each SH and equipment standards thus influenced the residents’ activity level and social function.

### Policy change can affect possibilities for activity

A consequence of a policy change in one municipality highlights the divide as shown above. The municipality withdrew the provision of cooked meals on the premises in order to stimulate greater self-dependence among the residents. The good intentions behind this policy decision resulted, however, in some residents only buying ready-made meals and eating alone. As seen above, food and its preparation have a variety of functions, and the staff face a dilemma in terms of individualizing this activity. They need to utilize their competence, skill and subtlety to balance the complex and contradictory nature of the activity and to be able to capitalize on the opportunities that this everyday situation provides, whilst not nullifying the important social aspects.

The skill set, which the staff need for practice, can be said to be similar to that found in milieu therapeutic environments, where the environment itself functions as a therapeutic agent and has the potential to affect therapeutic change [48]. Milieu therapy, with origins in residential treatment environments in the 1950s, is still relevant in similar contexts and can be related to more recent theoretical reasoning on how the environment can remotivate behavior [40, 49] or be modulated to create the right sensory stimulus [30, 31]. This can be an important area for the in-house training of SH staff.

### Discussion of method

The discussion of the rigor applied in the study is based on the principles of trustworthiness as presented in Graneheim et al. [28]. The data collection process contained a number of potential threats to the study’s trustworthiness. These included the participants’ difficulties with memory that resulted in blank spaces in the diary etc. The structure of the diary, the use of prompting questions and the confirmation from the participant that the diary was representative of an ordinary day, strengthened the credibility and trustworthiness of the study. The decision to process the diaries into a short narrative format enhanced the understanding by reorganizing the content in chronological order when needed and facilitated the analysis, thus contributing to ensuring credibility and confirmability. The authenticity was increased by the use of quotations. The diaries only cover 1 day and have limited information, and transferability is thus limited to similar settings. However, the large sample size and the structure of the data collection method helped to strengthen the credibility and transferability.

## Conclusions

Many residents in SH experienced loneliness and that they were offered few motivational opportunities to be active. Activities were mainly performed at home alone and basic needs were the main reason to go out into the community. However, the residents were also mostly satisfied with their calm and tranquil activities, saw no other options and appeared to demand very little of life, which has some similarities to the life of patients in former institutional environments. Several aspects of the results could be linked to the residents trying to find the right balance of activities in everyday life and the right amount of environmental stimuli. This needs to be considered in policy change and when planning and developing services.

Practice needs to become more recovery-oriented, to increase activity choices and interventions for residents in SH are warranted. In order to be more active and experience greater independence, the residents need to gain tools to cope with stimuli in the environment, to find more balance, and to gain motivation to be engaged in their own individual recovery journey. The fact that the role of the staff appeared to be important for enabling activity indicates that research is warranted on how staff perceive the support they give and how to enhance it further.

## Data Availability

The data supporting the conclusions of this article is available in the research groups repository.

## Abbreviations

SH: Supported housing
SMI: Severe mental illness
